# Exploring Non-Pharmacologic Adjunctive Therapies for Patients with Neurodegenerative Diseases

**DOI:** 10.3390/medicina61071224

**Published:** 2025-07-05

**Authors:** Maria Marchiș, Magdalena Iorga

**Affiliations:** 1 Faculty of Psychology and Education Sciences, “Alexandru Ioan Cuza” University, 700554 Iasi, Romania; maria.marchis@student.uaic.ro; 2 Department of Behavioral Sciences, Faculty of Medicine, “Grigore T. Popa” University of Medicine and Pharmacy, 700115 Iasi, Romania

**Keywords:** neurodegenerative disorders (ND), alternatives therapies, non-pharmacological therapies, neuromodulation therapies, neurocognitive therapy

## Abstract

*Background*: Alternative therapies, such as non-invasive neuromodulation techniques, cognitive therapies, virtual reality-based interventions, and psychological support, represent promising approaches for treating and supporting the management plan for patients with neurodegenerative disorders. Recent research has focused on the effectiveness of neuromodulation therapies, as they show improvements in both emotional and cognitive functions in patients with neurodegenerative disorders. *Material and Methods*: A literature review was conducted by searching Google Scholar, PubMed, Scopus, and Web of Science in February 2025. In total, 20 studies that met the inclusion criteria were considered for the present review, and the studies included were conducted between 2020 and 2025. *Results*: Innovative neuromodulation therapies have demonstrated their high potential in the management care plan of neurodegenerative disorders and as non-invasive neuromodulation therapies, both in the emotional manifestations of the patients and in the cognitive ones, with a direct impact on their caregivers’ experience. Although research is ongoing, the following preliminary findings are encouraging, suggesting that these methods may complement or even replace certain traditional interventions. *Conclusions*: Alternative therapies (non-invasive neuromodulation techniques, cognitive therapies, virtual reality-based interventions, and psychological support) represent promising approaches for treating and supporting the management care plan for neurodegenerative disorders, even in the cases where no other drug-based treatment option can be applied. Each method has its unique advantages, but further studies are needed to create treatment protocols and confirm their long-term effectiveness. Integrating these strategies into personalized management care plans can significantly improve cognitive function and emotional health and increase the quality of life of patients with cognitive and neurodegenerative disorders.

## 1. Introduction

Neurodegenerative disorders (NDs), as recent studies show, are a significant global health concern, given their increasing prevalence and the current limitations of the current pharmacological drug-based treatments, and they are the leading cause of disability and one of the highest rates of morbidity, with important impact on patients, caregivers, and society [1,2,3]. Dementia or Alzheimer’s disease (AD), Parkinson’s disease (PD), progressive supranuclear palsy (PSP), and other NDs are characterized by the gradual degeneration of neurons, which leads to progressive cognitive decline, motor impairment, and important neuro-psychological symptoms and manifestations that severely impair the patients’ quality of life (QoL). Disorders with autoimmune etiology, such as multiple sclerosis (MS) reflect neurodegeneration in their physiopathology and could benefit from non-pharmacological interventions [4,5]. People with these diagnoses can experience feelings of frustration, depression, anxiety, hallucinations, isolation, and meaningful reduction in their ability to engage in daily activities on their own [6,7]. To cover both the emotional and cognitive status of the patient, given the impact on emotional well-being and cognitive functions, NDs require special intervention that addresses both dimensions of the patients [8,9]. According to the World Health Organization (WHO, 2023, https://www.who.int/news/ (accessed on 1 March 2025)), the number of people living with NDs will triple by the year 2050, underlining the need for other kinds of interventions and therapies, as traditional pharmacological approaches primarily target the main symptoms of the disease rather than improving and managing the disease [10].

Although drug treatments have limited efficacy and also important side effects, psychological and non-pharmacological (alternative) therapies can play an important role in the management of these diseases. Recent research has focused on the effectiveness of neuromodulation therapies, as they show improvements in both emotional and cognitive functions in patients with NDs [11,12]. Innovative neuromodulation therapies have demonstrated their high potential in the management of NDs and as non-invasive neuromodulation therapies, both in the emotional manifestations of the patients and in the cognitive ones, with a direct impact on their caregivers’ experience.

Alternative therapies, such as neuromodulation therapies, have become a significant advancement in the treatment plan for NDs, particularly through non-invasive techniques such as Transcranial Direct Current Stimulation (tDCS), Transcranial Photobiomodulation (tPBM), Transcranial Magnetic Stimulation (TMS), Transcranial Pulse Stimulation (TPS) etc. [13,14,15]. As studies show, integrating these therapies into the treatment plan of patients with NDs can bring significant benefits to both emotional and cognitive status and also improve the impact on caregivers’ QoL. Although research is ongoing, the following preliminary findings are encouraging, suggesting that these methods may complement or even replace certain traditional interventions.

These interventions aim to focus on different aspects of the brain and behavior, as tDCS and TMS modulate neural activity and plasticity, with enhanced cognition and motor function, especially in conditions such as AD, PD, and MS. Unlike electrical or magnetic stimulation, tPBM works at the biochemical level by delivering near-infrared (NIR) light that penetrates the scalp and skull, which is absorbed by intracellular chromophores, most notably cytochrome c oxidase, a key enzyme in the mitochondrial electron transport chain. This stimulation enhances ATP production, reduces oxidative stress, and modulates cellular metabolism. These biochemical effects are thought to underlie the improvements in cognitive and emotional functioning observed in certain clinical contexts and also with effects on neuroinflammation and synaptic plasticity. Cognitive stimulation therapy (CST) and training is a low-tech but effective way to boost mental abilities and increase the QoL of patients with dementia and other cognitive disorders. Virtual reality (VR) therapy provides an engaging platform for motor and cognitive rehabilitation, and psychological approaches address the emotional status of these diseases, thereby significantly reducing depression, anxiety of the patient, and caregiver stress [16,17,18,19,20,21,22]. Implementing these therapies can yield significant benefits. CST and neuromodulation enhance memory and clear thinking, VR improves walking and independence, and counseling alleviates depression, helping patients and families find hope and resilience in progressive illness. Additionally, many of these therapies have synergistic potential; for example, combining non-invasive brain stimulation (NIBS) with cognitive training or psychological intervention might amplify each other’s benefits [16].

tDCS is a NIBS technique that delivers a weak constant direct current (usually between 1 and 2 mA) via scalp electrodes to modulate neuronal excitability; it is painless, easy to administer, and has no substantial side effects when used within standard parameters. tDCS has been studied as a therapeutic intervention for various NDs, including AD. Multiple randomized trials have indicated that tDCS may enhance cognitive performance in patients with AD. Additionally, in the context of PD, tDCS applied to frontal regions has demonstrated improvements in specific executive functions, such as increasing divided attention and verbal fluency and reducing cognitive interference susceptibility [16]. Studies have also explored tDCS for MS and found that it may ameliorate fatigue, pain, and cognitive slowing in MS patients. In progressive supranuclear palsy, studies have reported that a few sessions of tDCS over the prefrontal cortex can transiently improve language production in patients with PSP (phonemic fluency and naming ability), visual and motor coordination, processing speed, memory, and fluency after 10 sessions, suggesting that tDCS may have the potential to boost both cognitive and motor performance in PSP [23,24]. tDCS is generally regarded as safe when guidelines are followed. Minor side effects may include skin irritation under electrodes, slight tingling, or headache. It is advised to avoid tDCS for individuals with implanted electronic devices (such as deep brain stimulators or pacemakers), as the current could interfere with these devices. It is also contraindicated in patients with scalp lesions or dermatological conditions at the electrode points to prevent irritation, but overall, tDCS is a highly safe therapy [23].

TMS is a NIBS that is based on Faraday’s principle so that a high-intensity brief current passing through a coil is able to induce a magnetic field that will ultimately generate an induced current on brain circuitry, leading neurons to fire action potentials [17]. TMS has been explored both as a research tool (to probe cortical function) and as a therapy in neurodegenerative diseases, showing improved cognitive function, especially when applied to multiple brain sites involved in memory networks. It can lead to improvements in memory, language fluency, and overall cognition in AD and PD patients. TMS can enhance cognition (memory, attention, executive function) and provide emotional benefits (reduce depression, apathy) in neurodegenerative disorder patients. AD patients showed improved memory and language for weeks after TMS treatment. PD patients experienced better mood and motor functions, while MS patients saw improvements in fatigue and cognitive slowing [25].

Populations that may benefit most are those in mild-to-moderate stages of impairment—early AD or mild cognitive impairment, PD without dementia, MS with mild disability, and PSP before wheelchair dependence—as they have enough intact neural circuits for stimulation to engage and sufficient functional capacity. TMS is one of the few interventions that can directly reduce neuropsychiatric symptoms (such as depression) commonly accompanying neurodegenerative diseases, and depressed patients with PD, AD, or MS can see significant mood improvements from TMS [25].

tPBM involves delivering low-level red or near-infrared (NIR) light to the brain through the scalp or nasal passages to stimulate cellular and molecular processes that can protect or heal neural tissues. Unlike electrical or magnetic stimulation, tPBM works at the biochemical level, as photons of specific waves penetrate the scalp and skull and are absorbed by chromophores in cells and more within mitochondria [18]. The potential benefits of tPBM in these diseases include improvements in cognitive function, neuropsychiatric symptoms, and possibly in motor function or gait. For patients with AD, tPBM might lead to clearer thinking, better orientation, or improved short-term memory, while emotional and behavioral benefits have also been reported. Reduced anxiety, improved sleep quality, and more regular mood have been noted in patients with dementia undergoing tPBM, which significantly impacts QoL [18].

CST and cognitive training are non-pharmacological interventions that engage patients in mental exercises and activities to improve or maintain their cognitive function. Unlike previous neuromodulation techniques that directly focus on the brain physiology, CST works by improving the brain’s natural plasticity through practice exercises, as it typically involves, if not a special software designed for CST, a range of tasks such as puzzles, word games, reminiscence discussions, orientation exercises, or creative activities, and is usually a global stimulation of cognitive processes (memory, attention, language, reasoning) rather than intensive training of a single function [19]. Clinically, CST is recommended as a standard of care for people with mild-to-moderate dementia, which is the primary disease where CST was applied, with MMSE (Mini-Mental State Examination) scores around 10–24 [26]. CS therapies are advantageous because they are simple, safe, holistic, low-cost, and low-risk interventions. They often require trained facilitators, special computer programs for digital versions, or simple materials for traditional approaches, with no medical side effects [27].

VR therapy uses immersive computer-simulated environments to engage patients in interactive experiences that can be therapeutic, and with the help of VR headsets, patients can be placed in various virtual scenarios designed for rehabilitation or CST. VR can provide augmented feedback, repeatable exercises, and safe simulations of real-world tasks that may be challenging for patients in reality. The advantages of VR therapy include the high engagement level of the patient, the ability to simulate a wide range of scenarios from the real world, and the potential for objective performance tracking, which is often described as a motivating therapy, as patients may forget that they are “in therapy” because they are immersed in a game or virtual task [20]. There are no medical side effects, as it is not a direct method of therapy that targets the patient system.

Psychological approaches, such as various forms of psychotherapy and mental health interventions focused on the emotional, behavioral, and psychological challenges of individuals with NDs, including cognitive behavioral therapy (CBT), counseling, support groups, mindfulness interventions, and caregiver education or training in behavioral techniques. These approaches do not directly change the brain’s pathology, but they help patients and families cope with the illness, treat or help psychiatric symptoms (such as depression or anxiety) and optimize cognitive and emotional functioning through strategies that help patients reframe negative thoughts and engage in other types of pleasurable activities, often with modifications to accommodate cognitive limitations [28]. Psychological interventions are applicable across all neurodegenerative diseases, as each of them comes with significant stressors and often psychiatric comorbidities, and the benefits include improved mood (reduced depression, anxiety, and apathy), better coping and adjustment (less stress and more control), enhanced social interaction and communication, and indirectly maintained cognitive function via reduced psychological barriers [29]. The advantages of psychological approaches include their ability to address aspects of illness that medications and physical treatments often do not include: emotional suffering, coping, and behavioral management. They are holistic, improving mental health, which can positively affect physical health and cognitive functioning. The main limitation is the accessibility of these services, as many patients do not have easy access to a psychologist or therapist who understands neurological conditions. Another limitation is the cognitive impairment itself; therapy techniques such as CBT require memory, abstraction, and insight, which are low in moderate-to-severe dementia patients [21,28].

## 2. Materials and Methods

The present literature review aimed to synthesize findings from previous research that explored psychological interventions and alternative therapies for ND. We used electronic databases, such as Google Scholar, PubMed, Scopus, and Web of Science, to find the existing studies and literature, and we performed the review process through several steps, which are presented below. For the initial search, titles and abstracts were evaluated by reading the full articles, and the selected studies were added to Zotero.

### 2.1. Search Strategy

We used electronic databases such as Google Scholar, PubMed, and Web of Science to identify relevant literature on psychological interventions and alternative therapies for ND. Additionally, the existing studies were searched using a couple of combination of keywords such as “psychological interventions”, “alternatives therapies”, “neuromodulation therapies for neurodegenerative diseases”, “psychological and alternatives therapies for ND”, “ND” and related terms (the ND: Dementia or AD, PD, PSP, MS, and other neurodegenerative diseases). The present review was carried out in February 2025 and the studies included were conducted between 2020 and 2025.

### 2.2. Study Selection and Data Extraction

All included studies were initially selected based on their titles which included “psychological approaches for ND” and “alternatives neuromodulation therapies for ND” that were written in English. Then, the first analysis related to the paper’s abstract and full text was carried out to be evaluated according to the inclusion criteria of the present review. Data extraction was performed using a structured form to capture key information from each included study. Thus, data extraction followed several essential pieces of information, such as study objectives, methods, and key findings related to alternative therapies for neurodegenerative diseases.

### 2.3. Inclusion and Exclusion Criteria

In shaping our inclusion criteria, we focused on studies published from 2020 to 2025, which examined non-pharmacological approaches (like psychological interventions, neuromodulation, virtual reality, or cognitive training) applied in older adults with ND. Inclusion criteria were: studies involving participants aged 60 or older diagnosed with a neurodegenerative condition; interventions that were non-invasive and non-drug-based (e.g., tDCS, tPBM, psychotherapy, VR); outcomes reported in at least one cognitive (e.g., memory, attention) or emotional (e.g., depression, anxiety) domain; original interventional studies or systematic reviews with empirical synthesis. Exclusion criteria were: studies that focused only on drugs or surgical/invasive methods; research centered on caregivers or healthcare staff instead of patients; papers lacking concrete cognitive or emotional outcome data; conceptual or theoretical work without empirical evidence; samples unrelated to our target population (e.g., psychiatric or healthy controls); studies that lacked an intervention entirely or targeted only family dynamics; duplicates or non-peer-reviewed versions of articles already considered; with unclear methodology or not clinically applicable. In the end, 20 studies were selected that, in our view, supported the broader goals of this study.

- Records identified through databases: *n* = 9.770
- Records screened: *n* = 9.333
- Records excluded based on title, keywords and abstract: *n* = 8.820
- Full-text articles assessed for eligibility: *n* = 512
- Full texted articles excluded: *n* = 492
- Studies included in review: *n* = 20

## 3. Results

### 3.1. Study Selection

Studies specifically focusing on psychological interventions and alternatives therapies for ND were selected. In total, 20 studies that met the inclusion criteria were considered for the present review.

### 3.2. Study Characteristics

A total of 20 studies were included in our literature review (see Table 1). The studies were conducted between 2020 and 2025 in Germany (N = 2), Hong Kong, China (N = 3), Italy (N = 2), Iran (N = 2), Portugal (N = 2), the USA (N = 1), Canada (N = 2), Spain (N = 1), the UK (N = 1), Australia (N = 1), China (N = 1), Sweden (N = 1), and Brazil (N = 1).

### 3.3. TPS in Neurocognitive Disorders

The possibility of using TPS as a non-invasive intervention for elderly persons to enhance cognitive function was investigated by Lo et al. [31]. In this study, 17 patients underwent TPS sessions for two weeks, and cognitive tests and fMRI scans were conducted both before and after intervention. The results of the Montreal Cognitive Assessment (MoCA) showed notable alterations in functional connectivity within important brain regions, specifically in the hippocampus and parahippocampus, which were associated with enhanced global cognition. An inverse relationship between cognitive performance and cortical thickness in the right precuneus was found, indicating that TPS can alter brain connectivity to improve cognitive functioning even in the absence of major alterations in cortical thickness.

According to these results, TPS represents a potential complementary therapy for mNCD. Lo et al.’s [31] results demonstrated the safety of TPS therapy, as participants indicated just a little discomfort and no notable side effects of the procedure. TPS can have an important impact on neuronal networks, especially in the region of memory processing and the control of emotional symptoms, as evidenced by the alteration of functional connectivity in limbic structures, such as the hippocampus and parahippocampus, suggesting that TPS may promote neuroplasticity.

Another study, focusing on TPS, was the retrospective study by Cont et al. [32]. This study evaluated the use of TPS in individuals with mild-to-severe Alzheimer’s disease. Using scales such as the Alzheimer’s Disease Assessment Scale (ADAS), MMSE, and MoCA, cognitive and emotional functioning were evaluated in 11 patients who received TPS treatment. The results showed that changes in the MMSE and MoCA scores were not deemed statistically significant, but substantial improvements were observed in the ADAS total and cognitive subscale scores. Subjective reports from patients demonstrated substantial improvements in the emotional area (depressive symptoms and symptom intensity). Reported side effects were very mild. Research indicated that even individuals with moderate to severe Alzheimer’s could experience advantages from the TPS therapy, pointing to its possible role as a secure and effective supplementary treatment throughout various stages of the disease.

### 3.4. tPBM as a Neuromodulation Technique

The safety and initial effectiveness of tPBM in the treatment of moderate cognitive impairment (MCI) were assessed in a pilot study by Rashidi-Ranjibar et al. [38]. Fourteen participants with an MCI diagnosis were assigned to active or sham tPBM treatment in this randomized and placebo-controlled study. The study was conducted for six weeks, and at this time the participants received daily tPBM sessions. Executive functions, overall cognitive abilities, and brain biomarkers (determined by neuroimaging and blood-based indicators) were the main end measures, and when comparing the active tPBM group to the sham group, the results showed noteworthy improvements. The Trial Making Test-B (TMT-B) was completed with greater efficiency by the participants who received active tPBM, indicating improved executive cognitive function. According to neuroimaging results, the increase was in the right thalamic volume and functional connectivity within the limbic and default mode networks (DMN). These results indicate that tPBM therapy may improve neural connections, modify mitochondrial activity, and promote cognitive performance in patients with MCI.

Liebert et al. [39] evaluated the success of tPBM in reducing the clinical symptoms of PD through a prospective proof-of-concept study. Twelve individuals with idiopathic PD, who received transcranial, intranasal, neck, and abdominal tPBM treatment during a twelve-week clinic session, followed by a home treatment phase to maintain the results, participated in that study. Before, during, and after therapy, the participants’ mobility, cognitive abilities, fine motor skills, motor balance, and general QoL were evaluated using standard scale measures. Statistical significance was assessed using the Wilcoxon signed rank test. Following twelve weeks of tPBM treatment, the results showed significant improvements (*p* < 0.05) in the patients’ fine motor abilities, mobility, cognition, and balance, with many individual improvements above the smallest clinically detectable difference. Improvements lasted for up to a year during the home treatment phase, indicating that tPBM use can continue to have positive effects. No side effects were noted, demonstrating the safety and tolerability of this therapy. Despite identifying a possible Hawthorne impact, this study concluded that the treatment impact was significant and unique. The results showed that tPBM is a promising non-invasive treatment for PD that may help with a variety of clinical symptoms and improve the QoL in general. The preservation of benefits with the ongoing tPBM treatment underscored its promise as a complementary therapy, especially considering the progressive nature of PD, where symptom deterioration can be expected. Although some progress was observed, its methodological constraints (small sample size, *n* = 12) and the absence of a control group limit the strength of its conclusions. These findings, while encouraging, should be regarded as preliminary, and further research with more rigorous designs is needed to validate these observations.

The impact of tPBM on older people with MCI was also investigated in the study by Cheung et al. [33]. Over nine weeks, three participants with this diagnosis received 18 sessions of tPBM stimulation. The evaluation summarized standardized scales regarding anxiety and depression symptoms, global cognitive functions, everyday functioning skills, and neuropsychological tests (memory, attention, and executive function). The findings showed significant improvement in everyone’s frontal lobe cognitive abilities. In verbal memory and fluency tests, the subjects’ initial results were significantly impaired because of inhibited intrusion and perseverance errors. The tPBM intervention resulted in a significant reduction of these limitations, improving the ranges from the second percentile to the 83rd percentile. The study also identified a notable reduction in symptoms of depression and anxiety. The depression and anxiety symptoms of one participant improved from the mild to the normal range, while those of the other two participants fell from the severe to the mild range. Those outcomes suggested that tPBM can improve mental health and frontal lobe cognitive abilities in older people with non-amnestic MCI.

The effects of tPBM therapy on the cognitive status and psychological status in people with AD and mild cognitive impairment were also assessed in a pilot randomized controlled experiment by Razzaghi et al. [46], where thirteen people participated in a 12-week trial: six of the patients were placed in the tPBM group and seven in the sham treatment group. tPBM sessions, lasting 20 min daily, used an LED device emitting near-infrared light (810 nm). Measures included the Hamilton Anxiety Rating Scale (HAM-A), Hamilton Depression Scale (HAM-D), Disability Assessment for Dementia (DAD), and cognitive function (MoCA). The results of the tPBM group showed a statistically significant enhancement in functional ability levels (*p* = 0.041) compared to the control group. Although there was a trend toward improvement in the intervention group, no statistically significant differences were observed between the groups regarding cognitive or psychological outcomes. Therefore, the findings should be interpreted with caution as larger randomized controlled trials are needed to assess the cognitive efficacy of tPBM in AD.

### 3.5. Effects of Repetitive Transcranial Magnetic Stimulation (rTMS)

Espiritu et al. [40] comprehensively reviewed the effects and tolerability of rTMS as a treatment for people with traumatic brain injury (TBI), MCI, stroke, and neurodegenerative diseases. This analysis considered 14 studies with 418 participants, including 3 before-and-after investigations and 11 randomized controlled trials. Standardized measures such as the Neuropsychiatric Inventory-Apathy Subscale (NPI-AS), Apathy Evaluation Scale (AES), and Apathy Inventory (AI) showed a decrease in apathy. Findings also suggested rTMS improved AES and NPI-AS ratings, positively affecting apathy in AD patients across multiple studies. A single randomized controlled experiment in patients with primary progressive aphasia revealed that rTMS treatment significantly decreased apathy. There was conflicting evidence on the effects of rTMS on MCI; some studies showed no discernible changes, whereas others reported benefits. While one study on mild TBI found no significant correlation between rTMS and apathy decrease, a randomized trial in individuals with chronic stroke indicated possible improvements in patients’ symptoms of apathy. The review highlights that rTMS methods vary in target brain areas, frequency, and intensity, but no serious side effects were reported. The therapy was generally safe and well-tolerated. The conclusion of the study showed that rTMS had potential as a non-pharmacological treatment for apathy, especially in AD, but the authors pointed out the need for more extensive clinical trials to validate and identify the best stimulation parameters.

He et al.’s [42] randomized controlled trial investigated how intermittent theta burst stimulation (iTBS) affected the cognitive impairment in patients with mild cognitive impairment and PD. In this double-blind study, 35 individuals underwent sham stimulation (*n* = 15) or active iTBS (*n* = 20). Cognitive tests were conducted at three time points: prior to the intervention, immediately following the intervention, and three months after the intervention. The stimulation was applied to the left dorsolateral prefrontal cortex (DLPFC) for ten consecutive days. The primary outcome measures were the MoCA and Repeatable Battery for the Assessment of Neuropsychological Status (RBANS), with neuroimaging as supplementary outcomes. The results demonstrated that compared to the sham group, the iTBS group showed significant improvements in the scales (RBANS and MoCA) scores both at the three-month follow-up and immediately following treatment (*p* < 0.001). Additionally, improvements were observed in language processing, visuospatial skills, and immediate memory. Another potential biomarker for responsiveness to neuromodulation therapy was the neuroimaging data, which showed a positive correlation between baseline radiotracer uptake in the bilateral basal ganglia and cognitive improvement following iTBS. According to the study’s findings, patients with PD and MCI might experience sustainable cognitive improvements from iTBS therapy applied over the DLPFC. The authors also highlighted the necessity for larger-scale trials to improve treatment parameters and to assess the long-term efficacy of iTBS as a non-invasive treatment option for cognitive impairment in PD.

### 3.6. Effects of tDCS and Alternating Current Stimulation (tACS)

The effects of transcranial tDCS as a therapy for cognitive rehabilitation in individuals with PD and AD were investigated in a systematic review by Cammisuli et al. [16]. The research study comprised 17 randomized controlled studies (RCTs) assessing the impact of tDCS on executive functions, memory, and worldwide cognition; 10 addressed AD and 7 with PD. The MMSE, the ADAS-Cog, and other executive function tests were used in the final analysis. The findings showed that tDCS improved recognition memory and overall cognition in patients with AD. The temporal cortex was stimulated at 1.5–2 mA for 15–30 min, which improved verbal and visual recognition memory, and tDCS that addresses the DLPFC was associated with improvements in verbal fluency, divided attention, and a decrease in interference sensitivity in individuals with PD. However, the benefits of tDCS varied between studies across multiple cognitive areas, including working memory, processing speed, and visuospatial abilities, which underlined the variety in designs, including differences in patient groups, electrode location, session duration, and stimulation parameters.

tDCS was well tolerated and only caused minor side effects (tingling or soreness in the scalp in the first phases). The authors underlined that to create tDCS as a useful cognitive rehabilitation technique for AD and PD, larger RCTs with stimulation protocols are required.

The randomized, double-blind controlled study by Zhang et al. [41] aimed to determine whether tACS can improve the QoL for patients with PD. The main premise of this research was that tACS enhances motor and non-motor functions by modulating brain oscillations, thereby optimizing the outcomes of PD rehabilitation. A total of 60 individuals between the ages of 45 and 70 years who had Hoehn–Yahr scale scores between 1 and 3 were recruited for the trial and randomized to the tACS + multidisciplinary intensive rehabilitation treatment group or the sham-tACS + MIRT group. There was a 24-week follow-up period after the two-week intervention phase. The HAM-D, MoCA, and Non-Motor Symptoms Scale (NMSS) were used to evaluate non-motor symptoms. fMRI and EEG analysis investigated alterations linked to neuroplasticity. The central-peripheral-central rehabilitation concept, which states that enhancing brain connections and functional outcomes can be achieved by triggering neuroplasticity in central pathways, represents the foundation of the present study’s methodology. These results suggest that tACS may improve motor performance and cognitive abilities, thereby improving the general QoL. However, long-term effectiveness and ideal stimulation conditions have not yet been determined. The effects of recurrent prefrontal tDCS on mental health and cognitive impairment in patients with MP was examined in the study by Zakibakhsh et al. [34]. A total of 40 MS patients were enrolled in this present randomized, double-blind parallel-group study and were assigned to receiving either sham or active (1.5-mA) tDCS. Ten 20 min sessions were provided on alternate days to focus on the right frontopolar cortex (FPC) and left DLPFC. Quality of daily life, sleep issues, and psychological discomfort were the main end-measures, and neuropsychological tests evaluating psychomotor speed, working memory, and attention/vigilance were used to evaluate cognitive performance. The outcomes showed that in comparison to the sham group, the active group’s QoL was considerably enhanced by recurrent prefrontal tDCS, which also decreased psychological discomfort and sleep issues. Improvements in mental abilities were also observed in the vigilance, attention, and psychomotor speed. Improved cognitive performance was predicted by improved mental health outcomes. Prefrontal tDCS may be a promising non-invasive treatment for cognitive and mental health deficits in patients with MS, according to the study’s results. The study also demonstrated the safety and tolerance of tDCS. Patients reported very minor side effects (including a slight burning feeling). The authors suggested that tDCS-induced neuroplasticity, including brain areas linked to executive function, mood regulation, and cognitive control, may be responsible for the observed cognitive and emotional improvements. However, the authors suggest larger randomized controlled studies to verify these outcomes and evaluate the durability of tDCS effects in patients with MS.

Similarly, Satorres et al. [35] investigated the effectiveness of tDCS administered at home in individuals with moderate neurocognitive disorder (mNCD) and AD. An active tDCS group (*n* = 17) and a placebo group (*n* = 16) represented 33 participants in this randomly allocated, single-blind controlled-placebo research. Assessments were performed both at the beginning and after ten sessions of anodal tDCS (over the left DLPFC). Learning aptitude, prompt and postponed memory, and general cognitive function were the primary outcome measures, with executive function tests as supplementary end measures. Comparing the active tDCS group to the placebo group, the results showed a significant improvement in the learning capacity, immediate and delayed memory, and general cognitive functions. There was no significant improvement in executive function performance. These results are consistent with previous research indicating that tDCS improves memory and learning-related cognitive abilities but has no effect on executive functioning in patients with mNCD. The study also demonstrated the safety and viability of using home-based tDCS as a non-invasive treatment method for cognitive deficits in older adults. Only minor and temporary adverse effects (including a tingling feeling beneath the electrodes) were recorded by the participants, who tolerated the intervention well. More studies with larger samples and longer-term monitoring are required to determine how long cognitive gains last and to improve neurodegenerative disease treatment regimens.

### 3.7. Cognitive Stimulation Therapy (CST)

The efficacy of CST adjusted for the Italian population in treating mild-to-moderate dementia in seniors was investigated in a study by Carbone et al. [30]. Participants in this multicenter controlled study, conducted across 16 healthcare facilities, were randomly assigned to either an active control group or the CST-IT group. The MMSE and ADAS-Cog were utilized to evaluate general cognitive performance, and the Narrative Language Test was applied to evaluate the patients’ language abilities. Secondary objectives included assessing the QoL using the Quality of Life in Alzheimer’s Disease scale (QoL-AD), as well as evaluating mood and behavior through the Cornell Scale and the Neuropsychiatric Inventory (NPI). Daily functioning was measured using the Disability Assessment for Dementia (DAD). According to the outcomes of the present study, the CST-IT group’s MMSE scores were constant both at the three-month follow-up and immediately after the intervention, while the control group’s levels decreased. The ADAS-Cog and narrative language skills of the CST-IT group demonstrated notable gains that persisted over time. Neuropsychiatric symptoms (e.g., depression) worsened in the control group, while mood and behavioral symptoms did not worsen in the CST group. No appreciable alterations in daily functioning were noted, which is consistent with other studies indicating that the CST had little effect on fundamental daily tasks. The study results indicate that CST may serve as a non-pharmacological therapy in clinical practice, helping to maintain cognitive and emotional functioning while reducing behavioral symptoms in patients with dementia. This study demonstrated the value of early intervention by showing that CST had a greater positive impact on participants’ cognitive and affective areas when their baseline MMSE scores were higher. According to the aforementioned findings, applying CST in the early stages of dementia may increase its effectiveness and slow the onset of symptoms. In addition to supporting cognitive abilities, the format of CST sessions focused on social contact, which can sustain mood and reduce the neuropsychiatric symptoms. These results highlight the importance of incorporating the CST into dementia care management plans.

Gil et al.’s [43] quasi-experimental pilot study contrasted the efficacy of CST and recollection therapy (RT) for older persons experiencing cognitive deterioration. A total of 76 individuals were selected from Portuguese community support structures for this study. A total of 50 were assigned to the RT group and 26 to the CST group. The therapy sessions were performed twice a week for a period of seven weeks. MoCA, Geriatric Depression Scale (GDS-10), and WHOQOL-OLD-8 were used to evaluate cognitive performance, symptoms of depression and QoL. According to the findings both RT and CST significantly enhanced cognition, especially in the delayed recall capacity. Although there was a downward trend in depressive symptoms among the groups, the effect was not statistically significant at all. Furthermore, during the sessions, RT members showed greater involvement and improvement in communication, which facilitated more social interaction. According to the study’s outcomes, older adults who are experiencing cognitive decline can benefit from both RT and CST, with RT specifically enhancing their QoL. These findings suggest that reminiscence-based interventions can enhance well-being through personal narratives and social interactions.

### 3.8. VR in Neurorehabilitation

The efficacy of CST using a head-mounted display VR for neurorehabilitation was examined in a study by Specht et al. [36]. This randomized study included 42 participants. They were randomized to either the traditional computerized cognitive training (CCT) group (*n* = 21) or in a comprehensive VR training group (*n* = 21). To improve executive skills (such as organizing, focusing, and figuring out solutions) the VR intervention used a game format that included everyday life activities like cooking or even gardening. The principal result assessments involved cognitive tests such as the Trail Making Test (TMT), Tower of London (TL-D), and Wechsler Memory Scale (WMS-R). QoL, subjective health status, and affective measures were evaluated using predefined surveys constructed for this present study. The results showed that the VR training group significantly improved executive functions, especially planning and problem-solving tasks (*p* = 0.046, Bonferroni *p* = 0.02). Regarding the ability to pay attention to processing speed and memory, all participants showed signs of development, but they did not become statistically significant. In contrast, no discernible cognitive gains were demonstrated by the CCT group in any of the categories examined. Furthermore, although these results were not significantly different, participants in the VR group reported higher subjective well-being and health-related QoL indicators than those in the control group. According to the study’s findings, individuals undergoing neurorehabilitation can improve their executive skills more effectively with immersive VR cognitive training than with traditional computerized cognitive training. VR’s strong validity in the environment, which helps participants practice real-world duties in a virtual setting, could help improve cognitive abilities transferable to everyday life situations. The reviewers underline the requirement for more extensive studies with ongoing monitoring in addition to the present results to confirm the efficacy of VR-based cognitive training in neurorehabilitation and investigate its possible use in other ND.

Coelho et al. [47] studied the viability and the impact of using VR headsets to sustain memories in dementia. Nine patients with dementia participated in four recollection therapy sessions using 360° recordings of significant places from their lives (as a part of this mixed-methods approaches research). Participant involvement and behavioral and psychological symptoms were the main factors evaluated in this study. Furthermore, QoL and neuropsychiatric symptoms were assessed both before and after the intervention. Qualitative interviews were also conducted to learn about the experiences of their caregivers. The outcomes showed that the majority of participants enjoyed the 360° VR environment and immediately recalled their earlier experiences. A large percentage of the participants (57.7%) communicated spontaneously, giving personal memories of the places they visited. No significant increases in neuropsychiatric symptoms were noted (despite mild cases of agitation, anxiety, and irritability). There are very few reports of simulator sickness symptoms, including blurred vision and fullness of head. There were no statistically significant differences between the QoL and neuropsychiatric symptom assessments measured before and after the intervention. Caregivers noted improved participation and communication among participants, viewing the intervention positively. According to several caregivers, the immersive quality of VR made the recollection experience more significant for the participants. They also suggested some possible disadvantages, including the necessity of choosing clear and outstanding video material. In conclusion, this study found that VR-based recollection therapy is a safe and entertaining method for people with dementia.

### 3.9. Psychological Interventions and Other Approaches

Regarding the psychological interventions for depression and anxiety in people with dementia and cognitive impairment, the systematic review and meta-analysis by Orgeta et al. [37] included six randomized controlled studies evaluating the benefits of 439 participants with one diagnosis, which followed psychological interventions such as CBT, interpersonal therapy, supportive counseling, and multimodal interventions incorporating structured psychotherapy. First, the symptoms of depression and anxiety were measured, while secondary outcomes included QoL, cognitive function, ability of engaging in daily activities, and caregiver burden, suggesting that even if psychological treatments can reduce the symptoms, their impact on the patients’ daily functioning remain uncertain. Pharmacological treatments for emotional symptoms such as depression or anxiety in dementia have limited efficacy and potential side effects. An alternative is psychological intervention, which has shown benefits in managing care plans for dementia patients. Future research should focus on optimizing treatment delivery, evaluating long-term effects, and exploring the impact of psychological interventions on caregivers and overall patient QoL.

Kraepelien et al. [44] conducted a randomized study to evaluate how customized internet cognitive-behavioral treatment (ICBT) aids individuals with PD in improving their daily routines. A total of 77 patients participated in this trial. They were randomized to a waitlist control group that received only standard therapy or a 10-week ICBT program in addition to a normal care therapeutic plan. QoL, depression, anxiety, and sleeplessness were secondary objectives, while the overall functional impairment was the major outcome measured with Work and Social Adjustment Scale (WSAS). The results showed that the ICBT group had improved daily functioning compared to the control group and, with slight impact measurements, ICBT respondents indicated fewer symptoms of anxiety, insomnia, and depression. Only a third of ICBT attendees met the criteria for treatment responders, which was a 30% decrease in WSAS scores, and a large number of participants expressed high levels of involvement and satisfaction with the ICBT intervention, with the majority describing the program as reliable and helpful. According to the investigation findings, ICBT can help PD patients’ daily functioning and psychological health when used in conjunction with conventional medical treatment. To improve functional outcomes in PD care, future studies should examine long-term impacts, customized treatment adjustments, and integration with other rehabilitative treatments.

Gobbi et al. [48] study examined the effects of different types of physical activity on the psychosocial and cognitive abilities of 107 people with light to severe PD. Participants were divided into three intervention groups: multimodal (*n* = 38), functional mobility (*n* = 33), and mental/leisure (*n* = 36). Sessions were held twice a week for one hour each for 32 weeks. Psychological and cognitive tests were performed at baseline, four months, and eight months after treatment. Both Mental/Leisure and Functional Mobility training preserved cognitive function, particularly executive function, attention, and working memory. However, multimodal training did not halt cognitive decline in PD or provide significant cognitive improvements but did reduce physical stress after eight months of exercise. This study underlined the necessity of choosing suitable fitness programs that were adapted to the unique requirements of people with PD, as multimodal training might be more beneficial for physical well-being, and locomotor and cognitive training seemed to offer the greatest advantages for preserving cognitive health. Based on these results, organized, task-specific exercise may be extremely important in slowing the development of non-motor symptoms in PD and future studies should examine the long-term advantages of exercise therapies in greater detail and determine the best training regimens for enhancing cognitive and psychological results in people with PD.

Dobkin et al. [45] studied the effectiveness of telephone-based cognitive-behavioral therapy (T-CBT) in treating depression in patients with PD in a randomized controlled experiment. Seventy-two individuals with PD and clinical depression participated in the trial; they were randomized to either the T-CBT plus treatment-as-usual (TAU) group or the TAU-only control group. Ten weekly T-CBT procedures were performed, followed by monthly maintenance sessions for six months. The HAM-D was the main end assessment, and the HAM-A, the Short Form-36 for QoL, and the CBT goal engagement (reduction of negative thoughts) were secondary outcomes.

## 4. Discussion

Alternative therapies, such as non-invasive neuromodulation techniques, cognitive therapies, VR-based interventions, and psychological support represent promising approaches for treating and supporting the management plan for NDs. Each method has its unique advantages; however, further studies are needed to create treatment protocols and confirm their long-term effectiveness.

TPS is a promising non-invasive neuromodulation therapy for improving cognitive functions in patients with neurocognitive disorders, and studies such as the one conducted by Lo et al. underlined the potential of TPS therapy to modify brain connectivity, especially in key regions such as the hippocampus and parahippocampus, which are essential for memory and cognition. The results of this study suggest that TPS can improve cognitive function even in the absence of major structural changes at the cortical level, thus strengthening its role as a complementary therapy for minor neurocognitive disorders. Subsequent research, such as the study by Cont et al. [32], confirmed the effectiveness of TPS in improving cognitive and emotional functions in patients with mild to severe AD. Although some cognitive scales did not show statistically significant changes, notable improvements were observed in ADAS scale scores, and patients reported a better emotional state. The results of this study suggest that TPS can be beneficial at different stages of neurodegenerative disease, reducing symptoms (emotional or cognitive) and improving the QoL.

Another neuromodulation method, tPBM, has shown promising results in patients with mild cognitive impairment. Rashidi-Ranjibar et al. [38] observed significant cognitive improvements with increased thalamic volume and improved mitochondrial function. Liebert et al. also found that tPBM can improve motor function, balance, and cognition in patients with PD, indicating the potential of this therapy in the management of neurodegenerative conditions.

rTMS has also been investigated for its effects on neurorehabilitation, and the study of Espiritu et al. [40] showed that rTMS can reduce apathy in Alzheimer’s patients, while the study by He et al. [42] demonstrated that iTBS can improve cognition in people with Parkinson’s and MCI. These results suggest that rTMS is a promising non-pharmacological alternative treatment method, although further studies are needed to optimize stimulation parameters. Additionally, tDCS and tACS have also been explored for their effects on cognitive functions in patients with AD and PD.

Cammisuli et al. [16] suggested that tDCS therapy improved recognition memory and global cognitive performance in patients with Alzheimer’s, while Zhang et al. [41] found that tACS can improve motor function and QoL in PD. In addition to neuromodulation, CST was identified as an effective method for patients with dementia, and a study by Carbone et al. [30] demonstrated that CST can maintain cognitive function and emotional well-being in patients with dementia, with greater benefits when the intervention is applied in the early stages. Moreover, a study by Gil et al. [43] compared CST with RT and found that both methods improved memory and cognition, reinforcing the importance of structured cognitive engagement in patient care. Research on VR in neurorehabilitation showed encouraging results. Specht et al. [36] demonstrated that VR-based cognitive training improves executive functions more effectively than traditional methods, while Coelho et al. [47] highlighted the value of VR in reminiscence therapy for patients with dementia.

Consequently, the immersive nature of VR therapy can facilitate cognitive improvements and emotional status, transforming it into a promising alternative therapy for neurorehabilitation interventions. Psychological interventions have been effective in addressing depression and anxiety in patients with ND. Orgeta et al. [37] found that CBT and other psychological interventions can enhance emotional symptoms and QoL in individuals with dementia. Kraepelien et al. [44] demonstrated that i-CBT improved daily functioning and could reduce anxiety in patients with PD, while Dobkin et al. [45] showed that t-CBT was effective in reducing depression in these patients. These findings underline the importance of accessible psychological support for patients with ND that is complementary to medical treatments management plan. Additionally, structured physical activity was shown to be beneficial for maintaining cognitive and psychological symptoms and functions in ND. Gobbi et al. [48] found that functional mobility and mental training exercises helped preserve cognitive functions such as attention and working memory, suggesting that targeted physical exercises can slow the cognitive decline in these diseases. Given the progressive nature of PD, integrating tailored exercise into rehabilitation programs could significantly improve long-term outcomes.

Comparing the different brain stimulation techniques, as studies show, TPS can be more suitable for complex impairments (e.g., patients with advanced Alzheimer’s), as deeper brain activation is needed. For early stages, tPBM is usually preferred, targeting the mood symptoms and the mild cognitive decline. Also, tPBM can be used at home, and in early stages patients with good cognitive abilities can conduct the sessions by themselves. However, in cases where psychological distress, like apathy, or executive dysfunctions are predominant, rTMS can be used with good and promising results and targeted cortical modulation. tDCS also targets both emotional and cognitive status, and it can be chosen in disorders where both domains are affected, but in early to moderate stages, and is a viable therapy for maintenance, following initial intervention with rTMS. Details are presented in Table 2.

### Study Perspectives and Limitations

This literature review, by analyzing recent studies, highlights the clinical importance of the non-invasive neuromodulation techniques and their important role in addressing both cognitive and emotional symptoms of patients with ND.

From the perspective of biomedical science, our findings may support and provide a base for other further investigations into neuroplasticity, mitochondrial mechanism, neuromodulation and other similar mechanisms, contributing to a deeper understanding of their impact.

In clinical medicine, our findings may be a base of a multidisciplinary care model for the patients with NDs, with individualized non-pharmacological treatment plans incorporated. As some of these therapies are low risk and also highly accessible, they can also be applied even in home-based programs, which expand the medical settings with a lot of benefits for the patients and their caregivers. Also, combining these therapies (neuromodulation with psychotherapy or physical therapy) may offer holistic effects, resulting in optimized rehabilitation, especially in early and moderate stages of ND.

Several limitations should be considered, as our review is based on a wide range of studies, with different methodologies, stimulation protocols, and different pathologies and participants characteristics. Also, some of the clinical trials included in this review involved only small sample sizes, with a direct effect on the statistical data and conclusions. Future research should focus on larger randomized controlled trials and also on longitudinal studies in order to examine the cost-effectiveness of these potential non-invasive therapies and the sustained effects for patients with ND.

Despite encouraging findings reported immediately after intervention in most studies, only a small number followed participants over longer periods. As a result, it remains unclear whether the observed cognitive or emotional improvements persist in the long run. Moreover, potential safety concerns (particularly for at-home use of tDCS and tPBM) are not explored enough. Even though both methods are generally safe when applied correctly, errors in self-administration may lead to adverse effects (for example, misplacing electrodes or using high-intensity currents in tDCS can cause discomfort or skin damage, while unregulated use of tPBM devices might pose ocular or thermal risks). These gaps highlight the need for more detailed reporting on safety measures, follow-up outcomes, and clearer instructions when these techniques are used outside clinical settings.

## 5. Conclusions

Integrating these strategies into personalized management care plans can significantly improve cognitive function, emotional health, and the QoL of patients with cognitive and ND. Furthermore, the combined application of these therapies could have a synergistic effect, improving multiple aspects of the patients’ cognitive and emotional functioning. For example, combining therapies such as the rTMS with CBT or VR can increase the benefits of cognitive rehabilitation and reduce the emotional symptoms of anxiety and depression [49,50]. Similarly, integrating tDCS into a rehabilitation plan could improve both motor and executive function in patients with PD and other neurodegenerative conditions [51,52]. By combining neuromodulation therapies with psychological or cognitive interventions, we can obtain significantly superior results, beyond what one therapy can achieve alone, as non-invasive brain stimulation can modulate cortical excitability and also promote neuroplasticity, which supports the brain for more responsiveness to the therapeutic plan, creating a “window of enhanced learning” [50]. The simultaneous use of therapies capitalizes on biological, psychological, and cognitive status, supporting the patients more effectively and efficiently.

Thus, adopting multimodal therapies based on neuromodulation and psychological interventions can provide new perspectives on the management of cognitive and functional decline. In the future, the development of evidence-based clinical guidelines and personalization of these kinds of treatments according to individual patient characteristics will be essential to obtain an optimized therapeutic plan. Future application of artificial intelligence (AI) in neuromodulation therapies could be used for the selection of personalized stimulation parameters or techniques, based on individual patient characteristics, such as cognitive profile, neuroimaging investigations, diagnosis, and comorbidities. AI could support clinicians in identifying the optimal protocol for every case, and adjusting it in real time, resulting in more precise and accessible interventions. In addition, expanding access to these therapies through the use of digital technologies and home-based interventions could make them more accessible and sustainable in the long term. Therefore, an integrative, multidisciplinary, and patient-focused approach will be key to success in the treatment plan of ND. A multidisciplinary approach combining pharmacological and non-pharmacological therapies can provide the best results in slowing the progression of ND and improving the QoL of patients and their families.

## Figures and Tables

**Table 1:** Study characteristics.

	Author(s) and Year	Title	Study Characteristics	Country	Participants	Instruments Used	Diagnosis Target	Main Findings/ Relevance
1.	Elena Carbone et al., 2021 [30]	Cognitive Stimulation Therapy for Older Adults With Mild-to-Moderate Dementia in Italy	Assessment of the effectiveness of the Italian adaptation of the CST protocol on cognitive functioning and emotional symptoms in patients with dementia. Pilot study.	Italy	225	MMSE, ADAS-Cog, Narrative Language Test, Cornell scale, Neuropsychiatric Inventory, Disability Assessment for Dementia, Quality of Life Alzheimer Disease scale	Mild to moderate dementia	Italian CST adaptation improved cognitive and emotional symptoms.
2.	Heidi Ka-Ying Lo et al., 2024 [31]	Enhanced Cognition and Modulation of Brain Connectivity in Mild Neurocognitive Disorder	Examined the effects of Transcranial Pulse Stimulation on cognition and brain connectivity in mild neurocognitive disorders. Open label intervention.	Hong Kong, China	17	Cognitive evaluations, fMRI scans	Mild Neurocognitive Disorder	TPS enhanced brain connectivity and global cognition.
3.	Celine Cont et al., 2022 [32]	Retrospective real-world pilot data on transcranial pulse stimulation in mild to severe Alzheimer’s patients	Analyzed safety and short-term effects of TPS on Alzheimer’s patients across different severity levels. Retrospective study.	Germany	11	ADAS, MMSE, MoCA, Numeric Rating Scales	Mild to severe Alzheimer	TPS proved safe with short-term cognitive improvements.
4.	Mei-Chun Cheung et al., 2023 [33]	Photobiomodulation improves frontal lobe cognitive functions and the mental health of older adults with non-amnestic mild cognitive impairment	Investigated the effects of transcranial photobiomodulation on cognitive and mental health in older adults. Pilot study.	Hong Kong, China	3	Neuropsychological tests, standardized questionnaires	Mild cognitive impairment	Photobiomodulation improved frontal function and mental health.
5.	Nasim Zakibakhsh et al., 2024 [34]	Repeated prefrontal tDCS for improving mental health and cognitive deficits in multiple sclerosis	Evaluated the effects of repeated prefrontal tDCS on mental health and cognitive functions in MS patients. Randomized controlled trial.	Iran	40	MS quality of life, sleep quality, psychological distress measures, neuropsychological test battery	Multiple sclerosis	Reduced distress and improved cognitive performance.
6.	Encarnacion Satorres et al., 2023 [35]	Home-based transcranial direct current stimulation in mild neurocognitive disorder due to possible Alzheimer disease	Studied the effects of home-based tDCS on cognitive functions in mild neurocognitive disorder due to Alzheimer’s. Open label intervention.	Spain	33	Neuropsychological scales for cognitive function, memory, and executive functions	Alzheimer’s disease	Enhanced memory and executive functions at home.
7.	Julian Specht et al., 2023 [36]	Cognitive Training With Head-Mounted Display Virtual Reality in Neurorehabilitation	Examined the effectiveness of immersive VR cognitive training versus conventional computerized cognitive training in patients with stroke. Randomized controlled trial.	Germany	42	Wechsler Memory Scale, Trail Making Test A & B, Tower of London, Short Form 36, EQ-5D VAS	Post stroke	VR more effective than standard cognitive training.
8.	Vasiliki Orgeta et al., 2024 [37]	Psychological treatments for depression and anxiety in dementia and mild cognitive impairment	Evaluated the effectiveness of psychological treatments in reducing depression and anxiety in people with dementia and MCI. Systematic review.	UK	439	Various scales for depression, anxiety, quality of life, cognition, and caregiver burden	Dementia and mild cognitive impairment	Psychological therapy reduced anxiety and depression.
9.	Neda Rashidi-Ranjbar et al., 2024 [38]	A pilot study evaluating the feasibility, safety, and efficacy of transcranial photobiomodulation (tPBM) for the treatment of mild cognitive impairment (MCI): Preliminary findings	Investigated the efficacy of tPBM in improving brain functions in MCI. Pilot study.	Canada	14	Trail Making Test, Mini-Mental State Examination, MRI, fMRI	Mild cognitive impairment	Improved brain function and neuroimaging outcomes.
10.	Ann Liebert et al., 2021 [39]	Improvements in clinical signs of Parkinson disease using photobiomodulation	Assessed the effectiveness of tPBM in mitigating Parkinson’s disease symptoms. Open label study.	Australia	12	Mobility, cognition, balance, and fine motor skill assessments	Parkinson’s disease	tPBM improved motor and cognitive symptoms.
11.	Adrian I. Espiritu et al., 2023 [40]	Repetitive Transcranial Magnetic Stimulation for Apathy in Neurodegenerative Conditions: A Systematic Review	Reviewed the effectiveness of rTMS for treating apathy in neurodegenerative diseases, MCI, and TBI. Systematic review.	Canada	418	Cognitive and behavioral assessments	Neurodegenerative conditions, cognitive impairment, stroke, and traumatic brain injury	rTMS reduced apathy in ND and TBI.
12.	Davide Maria Cammisuli et al., 2022 [16]	Transcranial Direct Current Stimulation (tDCS) as a Useful Rehabilitation Strategy to Improve Cognition in Patients With Alzheimer’s Disease and Parkinson’s Disease	Evaluated the effects of tDCS on cognition in AD and PD patients. Systematic review.	Italy	17 RCTs (10 for AD, 7 for PD)	Neuropsychological tests, experimental cognitive tasks	Alzheimer’s disease and Parkinson’s disease	tDCS improved cognition in AD and PD.
13.	Hong yu Zhang et al., 2024 [41]	Transcranial alternating current stimulation improves quality of life in Parkinson’s disease: study protocol for a randomized, double-blind, controlled trial	Investigated tACS combined with rehabilitation for improving quality of life in PD. Randomized double blind trial.	China	60	Transcranial alternating current stimulation improves quality of life in Parkinson’s disease: study protocol for a randomized, double-blind, controlled trial	Investigated tACS combined with rehabilitation for improving quality of life in PD.	Protocol for improving QoL using tACS.
14.	Weijia He et al., 2021 [42]	Theta Burst Magnetic Stimulation Improves Parkinson’s-Related Cognitive Impairment: A Randomized Controlled Study	Evaluated the effects of iTBS on cognitive impairment in PD(PD) with MCI. Randomized controlled trial.	Hong Kong	35 (20 active, 15 sham)	Intermittent theta burst stimulation (iTBS) Repeatable Battery for the Assessment of Neuropsychological Status (RBANS) Montreal Cognitive Assessment (MoCA) 99mTc-TRODAT-1 SPECT brain scan	Parkinson’s	iTBS enhanced cognitive and neural activity.
15.	Isabel Gil et al., 2022 [43]	Effectiveness of Reminiscence Therapy versus Cognitive Stimulation Therapy in Older Adults with Cognitive Decline	Compared the effects of reminiscence therapy (RT) and cognitive stimulation therapy (CST) on cognitive decline. Controlled comparative study.	Portugal	76	Cognition tests, depressive symptomatology scales, quality of life measures	Cognitive decline	CST more effective than RT in cognitive outcomes.
16.	Martin Kraepelien et al., 2020 [44]	Individually Tailored Internet-Based Cognitive-Behavioral Therapy for Daily Functioning in Patients with Parkinson’s Disease	Evaluated the effectiveness of internet-based cognitive-behavioral therapy in patients with Parkinson’s disease. Randomized controlled trial.	Sweden	77	Work and Social Adjustment Scale, quality of life and depression measures	Parkinson’s	Internet CBT improved function and mood
17.	Roseanne D. Dobkin et al., 2020 [45]	Telephone-Based Cognitive Behavioral Therapy for Depression in Parkinson Disease	Investigated the effectiveness of telephone-based CBT for depression in patients with Parkinson’s disease. Randomized controlled trial.	USA	72	Hamilton Depression Rating Scale, anxiety and quality of life measures	Parkinson’s	Reduced depressive symptoms via phone CBT.
18.	Mohammadreza Razzaghi et al., 2024 [46]	Photobiomodulation’s potential as a non-invasive therapy for Alzheimer’s disease and minimal cognitive impairment: A 12-week investigation	Examined the impact of photobiomodulation on cognitive and psychological aspects in patients with Alzheimer’s disease and MCI. Open label pilot study.	Iran	13	Hamilton Anxiety and Depression Questionnaires, Daily Activity Questionnaire	Alzheimer’s and MCI	Reduced psychological distress, improved function.
19.	Tiago Coelho et al., 2020 [47]	Promoting Reminiscences with Virtual Reality Headsets: A Pilot Study with People with Dementia	Explored the feasibility and impact of using VR headsets for reminiscence therapy in people with dementia. Pilot study.	Portugal	9	Neuropsychiatric symptomatology and quality of life assessments	Dementia	VR feasible and beneficial for reminiscence.
20.	Lilian Teresa Bucken Gobbi et al., 2021 [48]	Effect of different types of exercises on psychological and cognitive features in people with Parkinson’s disease: A randomized controlled trial.	Compared functional mobility, multimodal, and cognitive training on cognitive function in patients with Parkinson’s disease. Comparative intervention study.	Brazil	107	Neuropsychological tests, cognitive function scales	Parkinson’s	Improved cognitive and psychological function.

**Table 2:** Comparative analysis between different brain stimulation techniques.

Brain Stimulation Techniques	Mechanism	Clinical Effects (from Included Studies)	Best Use Cases	Advantages	Limitations
TPS (Transcranial Pulse Stimulation)	Delivers focused mechanical shockwaves (ultrashort acoustic pulses)	- Improved global cognition and brain connectivity (Lo et al., 2024) [31]. - Safe across severe stages in AD (Cont et al., 2022) [32].	- Mild to moderate Alzheimer’s - Mild neurocognitive disorder	- Safe, tolerable in elderly - Rapid improvement of cognitive effects	- Specialized equipment - Limited accessibility
tPBM (Photobiomodulation)	Infrared light stimulates mitochondrial function and oxygen metabolism.	- Improved frontal lobe function and mood (Cheung et al., 2023) [33]. - Reduced anxiety, improved activity in MCI and AD (Razzaghi et al., 2024) [46]. - Symptom relief in Parkinson’s (Liebert et al., 2021) [39].	- MCI or early Alzheimer’s stages - Parkinson’s with mood symptoms and cognitive decline	- Home-based potential - Targets both cognitive and emotional status	- Less standardized - Small sample sizes in some studies
rTMS (Repetitive Transcranial Magnetic Stimulation)	Magnetic pulses target specific cortical areas (often DLPFC).	- Reduced apathy in ND (Espiritu et al., 2023) [40]. - Improved cognition in PD (He et al., 2021) [42].	- Parkinson’s with apathy or executive dysfunction - Mood/cognition in TBI or MCI	- Strong literature support - Precise stimulation	- Less tolerable for some
tDCS (Transcranial Direct Current Stimulation)	- Low-intensity electrical currents modulate cortical excitability. - Different protocols for anodal or cathodal stimulation.	- Improved cognition in Alzheimer’s and Parkinson’s (Cammisuli et al., 2022) [16]. - Reduced distress and improved QoL in MS and Alzheimer’s (Zakibakhsh et al., 2024 [34]; Satorres et al., 2023 [35]).	- Alzheimer’s, Parkinson’s, Mood Disorders. - Home protocols for early stages	- Low cost - Portable, feasible for home use - Can be delivered “online” (i.e., concurrently with a motor or psychological rehabilitative intervention) or “offline” (i.e., alone)	- Requires multiple sessions
